# Evaluating the Impact of a Photoaging App versus School-Based Educational Intervention on Adolescents’ Knowledge, and Attitudes towards Tobacco Use in Omani Public Schools: A Cluster Randomized Trial

**DOI:** 10.1192/j.eurpsy.2025.368

**Published:** 2025-08-26

**Authors:** T. Al-Mahrouqi, F. Al-Ghailani, M. Al-Maqbali, M. Al Saidi, P. Gouda Parameshwara

**Affiliations:** 1 Oman Medical Speciality Board; 2 Sultan Qaboos University; 3 National University of Science and Technology, Muscat, Oman

## Abstract

**Introduction:**

Tobacco use among adolescents remains a significant public health concern, particularly in the Middle East region.

**Objectives:**

This study compared the effectiveness of two interventions aimed at preventing tobacco use among Omani adolescents: the home use of photoaging app and a school-based educational module.

**Methods:**

In this randomized controlled trial, 188 adolescents were assigned to either the photoaging app or the educational module group.

**Results:**

The use of photoaging app demonstrated superior efficacy in enhancing perceptions of tobacco’s harmful effects, with 78.5% of participants recognizing tobacco as definitely harmful compared to 27.1% in the educational module group (p<0.001). Additionally, the app group showed greater resistance to peer pressure, with 95.7% stating they would “definitely” refuse tobacco if offered by a friend, versus 83.5% in the module group (p=0.031). However, the educational module was more effective in promoting support for smoking bans in public places. While not statistically significant, the photoaging app group showed a trend towards lower susceptibility to tobacco use.

**Conclusions:**

These findings suggest that integrating technology-driven interventions like photoaging apps with comprehensive educational programs could enhance tobacco prevention efforts among adolescents. Future interventions should consider a hybrid approach to leverage the strengths of both methods in combating youth tobacco use.

**Disclosure of Interest:**

None Declared

